# Dilute Polymer Droplets Show Generalized Wetting Dynamics via an Average Viscosity

**DOI:** 10.1021/acsapm.4c02170

**Published:** 2024-09-24

**Authors:** Amir Azimi Yancheshme, Heedong Yoon, Giuseppe R. Palmese, Nicolas J. Alvarez

**Affiliations:** Chemical and Biological Engineering, Drexel University, Philadelphia, Pennsylvania 19104, United States

**Keywords:** non-Newtonian wetting dynamics, droplet spreading, dynamic contact angle, shear thinning, dilute polymer solution

## Abstract

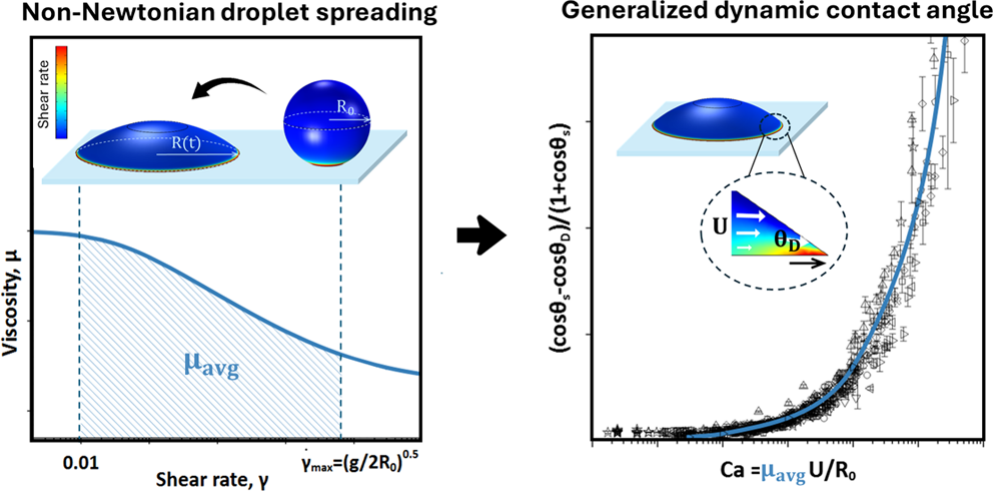

Despite the prevalence of non-Newtonian fluids in various practical applications, comprehensive dynamic wetting models are lacking. Existing models often oversimplify complex rheological behavior, limiting our ability to predict wetting dynamics. This work introduces and experimentally validates a generalized model for the dynamic wetting of non-Newtonian shear-thinning fluids (dilute polymer solutions) on solid substrates. We experimentally analyzed 12 different shear-thinning fluids using both a power-law model and Carreau–Yasuda model. The data clearly show that the dynamic contact angle can be generalized using an average viscosity to capture rheological changes during droplet spreading. The average viscosity was defined using the fluid’s constitutive model over shear rates relevant to the spreading process. Using a small droplet approximation, we propose and validate a semianalytical spreading model to predict the basal radius of non-Newtonian droplets. The model agrees well with the experimental data. Additionally, the average viscosity was used to define a spreading time scale, which is capable of collapsing the spreading of different non-Newtonian fluids onto a master spreading curve. This work offers significant potential for predicting the dynamic shape and spreading of non-Newtonian fluids with complex rheologies in a range of applications and industrial processes.

## Introduction

The dynamic wetting and spreading of non-Newtonian fluids, such as polymer solutions and colloidal suspensions, are critical in various industrial processes such as additive manufacturing, coating, functional surfaces, and microfluidics.^1−5^ For instance, in applications like inkjet and extrusion-based 3D printing, the wetting and spreading behavior of ink on substrates determines print resolution and dimensional accuracy.^6^ One of the primary challenges in the above applications remains the precise control of fluid spreading dynamics.^7^ Developing models to describe these dynamics is essential to enhancing the predictability and control of these processes. Unfortunately, there is a significant gap in the literature regarding the spreading of non-Newtonian fluids, and there exists no predictive non-Newtonian spreading model. This work proposes a simplified approach to model and predict the dynamic wetting of non-Newtonian fluids.

Dynamic wetting is a complicated process since it depends on surface tension, density, fluid–substrate interactions, volume, and fluid rheology.^8−10^ Due to this complexity, most studies are predominantly limited to Newtonian fluids. The spreading dynamics of Newtonian fluids has been thoroughly studied experimentally, with theoretical approaches and numerical simulations.^9,11−15^ Only a few experimental and theoretical studies address the spreading dynamics of non-Newtonian fluids,^1,16^ and these are mostly limited to simple inelastic power-law fluids, i.e., ,^8,17−25^ where  is the stress tensor, *n* is the power law, κ is the flow consistency index, and |γ̇| is the magnitude of the strain-rate tensor. It has been experimentally shown that the spreading exponents for shear-thinning fluids are less than those of Newtonian fluids, i.e., less than Tanner’s law (<0.1), with a spreading exponent dependent on the fluid’s shear-thinning rheology.^26,27^ The major challenge in modeling the spreading of non-Newtonian fluids is the boundary condition governing the motion of the contact line on the solid substrate.^4^ In our previous work, we showed that this issue can be overcome for Newtonian fluids by using a generalized dynamic contact angle model that depends only on the capillary number, Ca.^9^

There are two main approaches in the literature to address the contact line for non-Newtonian fluids: the molecular kinetic theory (MKT) and hydrodynamic (HD) approaches.^1,16^ MKT models are based on the Eyring rate process theory^28^ that introduces a molecular hopping mechanism at the contact line with frictional dissipation as the dominating channel of energy dissipation.^4^ MKT involves several adjustable parameters, such as (i) distance between two neighboring absorption sites on the solid surface and (ii) equilibrium frequency resulting from molecular displacements that are obtained by curve-fitting to experimental data of dynamic wetting.^4,29−31^ The HD models account for energy dissipation caused by viscous flow within the liquid wedge by solving the Navier–Stokes (N–S) equation and determining the velocity profile at the droplet’s edge, either theoretically through lubrication theory^3,15,17−20,24,25,32−34^ or via numerical simulation.^35^ The singularity at the contact line is mostly avoided by introducing a microscopic cutoff length, which serves as a fitting parameter when comparing with experimental results. Other microscopic approaches have also been suggested to address the nonintegrable stress singularity at the contact line, such as the existence of precursor films,^36^ diffuse interface,^37^ and shear thinning behavior near the contact line preventing divergence of energy dissipation.^38,39^ Details of each mechanism can be found in the review.^1^

HD and MKT models operate on different length scales and mechanisms of energy dissipation. Consequently, to leverage the benefits of both, several combined models have been developed.^40−42^ Nevertheless, it has been argued that these combined models yield similar fitting parameters to those of the MKT and HD models.^43^ It should be noted that these models are highly beneficial as they not only offer qualitative predictions about the dynamic wetting behavior but also shed light on the submicron features of fluids using macroscopic experimental spreading data. For instance, it has been proposed that the jump length in MKT gives an estimation of the Kuhn segment length of polymer chains.^4,44^ Overall, these two models are limited to fitting parameters that are fluid- and system-specific and are not capable of generalizing non-Newtonian spreading dynamics.^4^

To overcome this limitation in HD models, there have been several attempts to remove the fitting parameters via geometrical assumptions. For example, Carre and Eustache^19^ assumed a 2D wedge-like geometry near the contact line to analytically relate the contact angle, basal radius, and height. By comparing the magnitude of the cutoff length and basal radius for shear-thinning fluids, the cutoff length was removed from their final dynamic contact angle expression.^19^ Similarly, Liang et al.^18^ used a 3D cone-like domain to eliminate the cutoff fitting parameter and argue that the dynamic contact angle is insensitive to the cutoff length when *n* ≤ 0.6. Liang et al. tested their model against two non-Newtonian fluids and found them to be satisfactory. Although these models have been tested against limited experimental data, it is unclear that they can be used for a broad range of non-Newtonian fluids since they are limited to fluids with power-law behavior.^16^ Although Min et al.^45,46^ suggested that the work of Liang et al. can be adapted to nonpower-law fluids using equivalent power indices, it is not clear how such equivalent power indices would be determined.

The dynamic spreading of non-Newtonian, nonpower-law fluids is rarely investigated. One example combined the HD model with a simplified thixotropic constitutive model, referred to as the “1.5D model”.^47^ This model incorporates a structure parameter that accounts for both the buildup of the structure through Brownian motion and its breakdown due to shear. Although no comparison to experimental data was presented, the model suggests that thixotropy significantly affects the spreading dynamics of droplets. Another example incorporated a simplified Phan–Thien–Tanner constitutive model for viscoelastic fluids into the HD model.^2^ The model necessitates detailed rheological characterization, including measurements of relaxation time and an extensibility parameter. The model was compared to the spreading of one fluid and showed a relatively good agreement. Moreover, the spreading dynamics of thermoplastic polymer melts with complex rheology and reactive networks have been studied using both HD and MKT approaches.^44,48^ While model parameters need to be fitted for each specific fluid, the models provide valuable insights into the polymer chain/substrate interactions during spreading and the extent of polymer drop spreading.

In this work, we use experimental data for a range of non-Newtonian fluids to develop a dynamic wetting model that captures the dynamic contact angle and basal radius. The distinction of this work from the existing literature is a semianalytical model that depends only on measured material parameters, is not limited to a single constitutive relationship, and can be easily integrated to predict the spreading of non-Newtonian fluids with no fitting parameters. The paper is organized as follows: First, we characterize the rheology of a series of dilute polymer solutions. We then experimentally quantify the basal radius and dynamic contact angle as a function of time. An average viscosity is used to demonstrate a general scaling theory for the dynamic contact angle as a function of the capillary number. We propose and validate a semianalytical spreading model to predict the basal radius of non-Newtonian droplets. The semianalytical model is shown to be in good agreement with experimental data. Finally, we propose a modified viscous time scale for shear-thinning fluids, which can be used to generate master spreading curves that depend on the Bond number, Bo, and steady-state advancing contact angle, θ_*s*_.

## Materials and Methods

### Dilute Polymer Solutions

Xanthan gum (Sigma-Aldrich, CAS number: 11138-66-2) and carboxymethylcellulose sodium, CMC, (Sigma-Aldrich, CAS number: 9004-32-4) were dissolved in water and glycerol (RPI Research Products International, CAS number: 56-81-5)/water solutions to generate a series of test fluids. More specifically, three different types of test fluids were formulated: 0.3–0.5 wt % xanthan gum in DI water, 0.1–0.5 wt % xanthan gum in 60:40 glycerol/water solution, and 0.6–1 wt % CMC in DI water. We also used the reported drop spreading data in the literature (i.e., dynamic contact angle and basal radius versus time) of 0.1–0.4 wt % CMC in DI water,^45^ acrylic typographic ink,^19^ and 0.05–0.2 wt % xanthan in DI water^21^ along with our measured experimental data to further validate our models and simulations. Table 1 summarizes the fluids’ properties including density (ρ), surface tension (σ), steady-state advancing contact angle (θ_s_), initial size (*R*_0_), and Bond number (Bo = Δρ*gR*_0_^2^/σ) of each fluid.

**Table 1:** Fluid Properties and Wettability Conditions Used in Experiments and Those Extracted from the Literature

fluid	ρ [kg/m^3^]	σ [mN/m]	θ_S_ [°]	*R*_0_ [mm]	Bo
0.05 wt % xant.^21^	998	66	0	0.98	0.14
0.2 wt % xant.^21^	998	48	0	0.98	0.19
0.3 wt % xant.	998	47	10.9	0.95 ± 0.05	0.19
0.5 wt % xant.	998	45	11.2	1.15 ± 0.04	0.29
water/gly + 0.1 wt % xant.	1133	56	12.5	1.25 ± 0.06	0.31
water/gly + 0.3 wt % xant.	1133	56	12.9	1.22 ± 0.07	0.29
water/gly + 0.5 wt % xant.	1133	56	13.1	1.09 ± 0.03	0.23
0.1 wt % CMC^45^	1012	72	0	0.9–1.05	0.13
0.4 wt % CMC^45^	1012	71	0	0.9–1.05	0.14
0.6 wt % CMC	1012	64	14.5	1.12 ± 0.03	0.19
1.0 wt % CMC	1012	57	15.1	1.13 ± 0.02	0.22
acrylic typographic ink^19^	−	28	21.6	0.28	–

### Fluid Characterization

The steady shear viscosity (flow sweep) of each sample was measured using a DHR-3 rheometer (Waters/TA Instrument) with parallel plates and a gap height of 700 μm. The thixotropic properties of each solution were assessed using the hysteresis area method, which involved measuring the shear viscosity in fast data sampling mode (2 s per data point). The shear rate was increased from 0.01 to 1000 s^–1^ and then decreased back to 0.01 s^–1^ and measured at five rates per decade. The density and surface tension of all tested fluids were measured using a densiometer (DMA 501 Anton Paar) and pendant drop method with a contact angle goniometer/tensiometer device (ramé-hart instrument co./Model 90), respectively.

### Quantification of the Droplet Spreading and Dynamic Contact Angle

We used a contact angle goniometer/tensiometer device (ramé-hart instrument co./Model 90) to deposit and monitor the spreading of each fluid onto a microscope glass slide (AmScope). A pendant drop of approximately 4–15 μL was generated at the tip of either a 20, 25, or 27 gauge blunt hyperdermic needle using a syringe pump (HARVARD Apparatus, PHD 2000). The pendant drops were deposited gently onto the glass slide substrate by slowly lowering the droplet onto the surface using a *z*-axis stage, minimizing the influence of inertia. The calculated Weber numbers for the test fluids, ranging from 0.07 to 0.14, confirm that inertia is negligible compared to surface tension forces (see Supporting Information S2). To ensure the reproducibility of data, the microscope slides were washed and rinsed with isopropyl alcohol (VWR, CAS No. 67-63-0) and DI water (EMD Millipore Corporation) before each test. Each experiment was performed approximately nine times using a freshly washed slide. The accuracy of the optical system is validated before each experiment by confirming the surface tension of deionized water using the pendant drop method. The droplet was imaged from the side, and the basal radius and dynamic contact angle were extracted from the video recordings using Ossila Contact Angle v4.1.4 image analysis software (Ossila Ltd.). The contact angle is determined by a second-order polynomial fit to the droplet profile near the three-phase contact point by using the outer region of the interface. The contact angle is determined by an evaluation of the gradient calculated at the baseline.^49^ Another often used method to measure the contact angle is the fitting of the Young–Laplace equation to the droplet shape.^49^ We show in Figure S2 that the Young–Laplace (YL) method does not lead to significantly different values of the dynamic contact angle. The accuracy of the polynomial method was measured by the root-mean-square-error (RMSE) calculated locally near the three-phase contact line and ranged between 0.2 and 0.6 for all experiments (see Supporting Information S1). Note that the largest errors are measured at short time, when the angle is greater than 90°. See Supporting Information Figure S1 for an example image and error analysis. We monitored the volume of the deposited droplet throughout the spreading process, which remained consistent across all fluids, indicating that there was no evaporation.

## Non-Newtonian Dynamic Wetting Model

The key to modeling the spreading of any droplet is a good description of the three-point contact line boundary condition. We previously showed that Newtonian fluids follow a generalized scaling of dynamic contact angle (θ_D_) versus capillary number (Ca = μ*U*/σ) with the general form
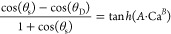
1where *U*, σ, and μ are the contact line velocity of the interface, surface tension, and viscosity, respectively, and *A* and *B* are experimentally determined parameters with values of *A* = 7.3 and *B* = 0.703 for a wide range of Newtonian fluids studied.^9^ For non-Newtonian fluids, the varying viscosities during droplet spreading complicate the definition of the Ca number. We propose that non-Newtonian fluids follow the same functional form as Newtonian fluids, albeit with different values of *A* and *B* when the capillary number is defined in terms of an average viscosity
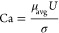
2where μ_avg_ is defined as the shear rate average viscosity given by
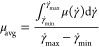
3The validity of eqs 1–3 will be tested by using experimental data.

Previously, we used eq 1 as a boundary condition in a full numerical simulation of the fluid mechanics of spreading.^9,10^ However, when the Bond number is small, there is a direct relationship among the dynamic contact angle, basal radius, and volume of the drop. Thus, eq 1 can be rearranged in terms of the contact line velocity, *U*, to obtain an ODE for *R*(*t*)
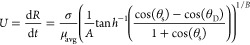
4such that there are two unknowns *R* and θ_D_. Equation 4 can be solved for the small Bond number approximation, which restricts the shape of the drop to a spherical cap, such that the volume of the drop, *V*_0_, is given by^50^
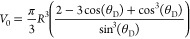
5Thus, *R*(*t*) and θ_D_ are predicted by independently measuring μ_avg_, σ, and θ_s_ and simultaneously solving the differential-algebraic system of equations (DAE spreading model). These equations were solved using “ode15i”, a built-in Matlab (Mathworks, USA) solver. For all cases, an initial contact angle of θ_*i*_ = 170° and its corresponding *R*(0) using eq 5 were considered for initial conditions. This DAE spreading model was tested against the series of non-Newtonian fluids described above. Note that solving the DAE spreading model resulted in the evolution of both *R*(*t*) and θ(*t*) over time. Additionally, one can obtain the height of the droplet with time by considering the geometric constraint of a spherical cap, i.e., *h*(*t*) = *R*(*t*)tan(θ(*t*)/2).

## Results and Discussion

The DAE spreading model presented above requires validation of several assumptions, namely, the ability to generalize the dynamic contact angle of non-Newtonian fluids via an average viscosity and the small Bond number approximation in eq 5. Furthermore, the model must be validated with experimental data to confirm its ability to accurately predict the spreading radius for a wide range of non-Newtonian fluids.

### Rheology of Test Fluids

**Figure 1:**
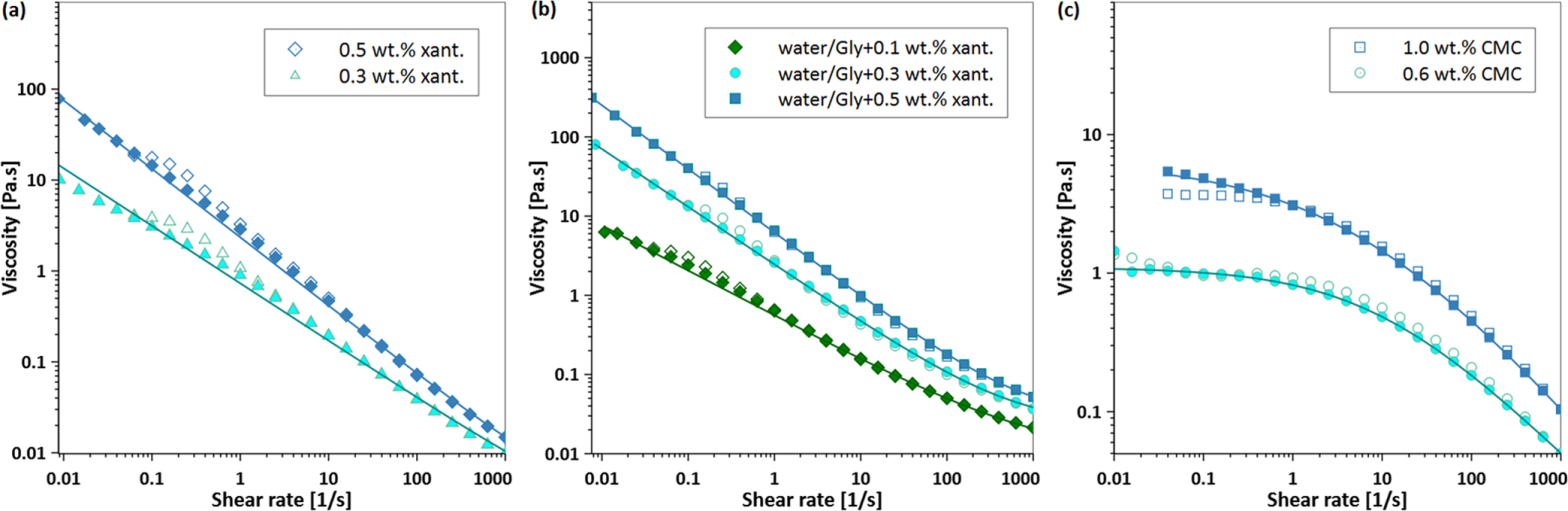
Rheology of test fluids and the corresponding rheological models.

Figure 1 shows the forward and reverse flow sweeps for xanthan, xanthan–glycerol, and CMC solutions at different concentrations. The hollow points and filled points represent forward and backward shear flows, respectively. As can be seen, all of the solutions show a very small hysteresis area, reflecting weak to no thixotropy. All of the test fluids including, xanthan, CMC, and xanthan–glycerol solutions are represented by the Carreau–Yasuda model, given by
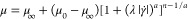
6where μ_0_ and μ_∞_ are the zero-shear and infinite-shear viscosity, respectively, λ is a time constant related to the relaxation time of the fluid, and *a* is the Yasuda parameter, which adjusts the transition region between the zero-shear viscosity and the power-law region. The best-fit parameters are shown as solid lines in Figure 1a–c and are given in Table 2. Table 3 also presents the best-fit parameters for the power-law model (μ = κ|γ̇|^*n*–1^), i.e., power-law index, *n*, and consistency factor, κ, for the sample fluids including xanthan solution and acrylic typographic ink, which were obtained from the literature.^19,21^

**Table 2:** Carreau–Yasuda Model Parameters for Xanthan, CMC, and Xanthan–Glycerol Solutions

fluid	μ_0_ [Pa.s]	μ_∞_ [Pa.s]	λ [s]	*a*	*n*	μ_avg_ [Pa.s]
0.3 wt % xant.	91.801	0.0012	2077.419	5.207	0.367	0.129
0.5 wt % xant.	396.341	0.002	908.565	5.207	0.246	0.359
water/Gly + 0.1 wt % xant.	50.933	0.010	2805.483	5.207	0.429	0.128
water/Gly + 0.3 wt % xant.	362.829	0.023	943.204	5.212	0.269	0.493
water/Gly + 0.5 wt % xant.	989.359	0.028	549.547	5.212	0.195	1.043
0.1 wt % CMC^45^	0.049	0.001	0.015	0.671	0.413	0.033
0.4 wt % CMC^45^	0.323	0.001	0.053	0.734	0.444	0.170
0.6 wt % CMC	1.110	0.001	0.098	0.550	0.343	0.351
1.0 wt % CMC	6.515	0.001	0.137	0.397	0.209	1.093

**Table 3:** Power-Law Model Parameters for Xanthan Solutions^21^ and Acrylic Typographic Ink^19^

fluid	κ [Pa.s^*n*^]	*n*	μ_avg_ [Pa.s]
0.05 wt % xant.^21^	0.033	0.629	0.011
0.2 wt % xant.^21^	0.284	0.437	0.058
acrylic typographic ink^19^	16.80	0.62	4.241

### Dynamic Contact Angle Model

**Figure 2:**
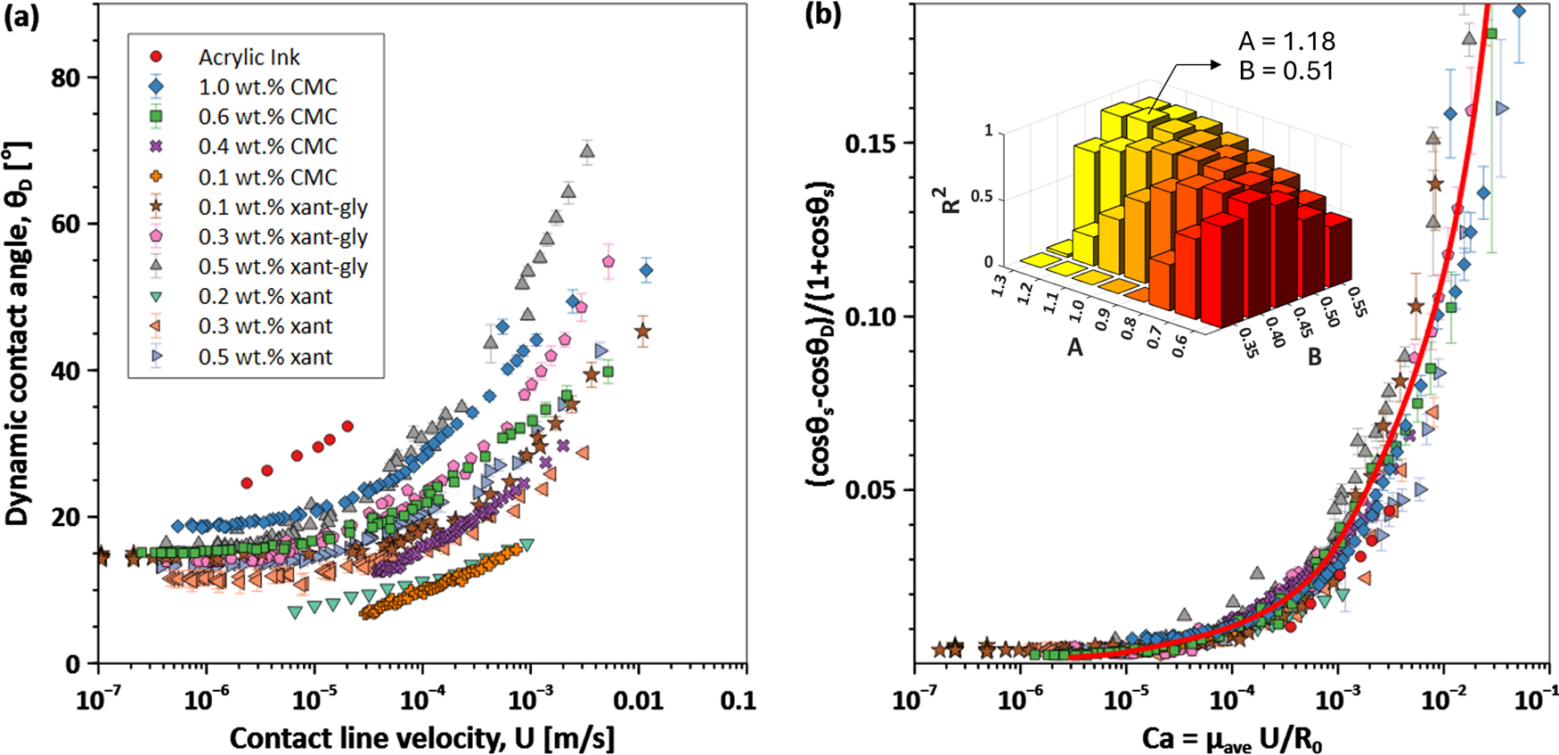
(a) Dynamic contact angle data versus the contact line velocity for xanthan solutions, CMC solutions, and xanthan–glycerol solutions measured in this study as well as the extracted experimental data of 0.2 wt % xanthan and acrylic typographic ink reported in (21) and (19), respectively. (b) Normalized dynamic contact angle data versus Ca number via the average viscosity. The inset displays the sensitivity analysis, illustrating the relationship between the calculated *R*^2^ and the model parameters *A* and *B* (eq 1). The optimal values identified are *A* = 1.18 and *B* = 0.51, which correspond to an *R*^2^ value of 0.94.

A dynamic contact angle model is essential for accurately predicting the spreading behavior in terms of *R*(*t*) through either theoretical or numerical simulations.^9^Figure 2a shows θ_D_ as a function of *U* for all test fluids reported in Table 1. As expected, each test fluid has a unique functional form based on its unique rheology. As shown in eq 1, the scaled dynamic contact angle is described by a relation between θ_D_ and the Ca number.^1^ For Newtonian fluids, this relationship works quite well when the Ca number is defined by the constant shear viscosity. Unfortunately for non-Newtonian fluids, the viscosity is not constant and varies as the droplet spreads, complicating the definition of the Ca number. The modified Ca number in eq 1 is defined in terms of an effective viscosity μ_avg_; see eq 3. The determination of μ_avg_ requires averaging the fluid’s constitutive model and specification of the shear rates corresponding to the limits of integration.

Given the axisymmetric geometry of our fluid system, the gradient of the radial velocity in the *z*-direction (γ̇_*zr*_) dominates the rate of deformation tensor near the contact line, especially at the late stage of spreading, such that the magnitude of the rate of deformation tensor is approximately the magnitude of the shear rate (second invariant) near the contact line, i.e., |γ̇| ∼ |γ̇_*rz*_|. As spreading progresses, the shear rate near the contact line steadily decreases, reaching minimal values as the equilibrium approaches. Thus, the end of spreading corresponds to a very low shear rate. Since spreading occurs on times less than 100 s, we restrict ourselves to a lower limit of integration of γ̇_min_ = 0.01 s^–1^, corresponding to the very late stage of spreading. A sensitivity analysis was performed to determine the effect of the lower limit cutoff on the average viscosity; see Supporting Information Figure S3. This figure clearly shows that the average viscosity is a weak function of the cutoff shear rate, and therefore, our lower limit is appropriate. If the dynamics of spreading occurred over much longer times and the average viscosity did depend strongly on the lower limit cutoff, then, a smaller lower limit may be applicable.

For the upper limit, a characteristic shear rate corresponding to the initial stage of spreading is considered. In spontaneous droplet spreading, the early stage is predominantly governed by gravitational forces rather than surface tension and edge effects. This could be why a simple energy balance considering only inertia is a good approximation to the upper limit in shear rate used to calculate the average viscosity, i.e., γ̇_max_ = (*g*/2*R*_0_)^0.5^, as detailed in Supporting Information S2. Note that while shear rate varies with spreading time, viscosity depends only on shear rate. Therefore, viscosity variation over spreading time can be completely captured by the dependence of shear rate with time. In other words, the viscosity is an implicit function of time. Integrating viscosity from the minimum to maximum shear rate is analogous to integrating from *t* = 0 to the end of the spreading process. μ_*avg*_ values for all test fluids are reported in Tables 2 and 3. Figure 2b shows that all fluids follow a master curve when the scaled dynamic contact angle is plotted as a function of the modified Ca = μ_avg_*U*/σ, similar to the result presented for Newtonian fluids.^9^ We used the generalized reduced gradient (GRG) nonlinear fitting algorithm^51^ to optimize model parameters by maximizing the coefficient of determination (*R*^2^). The inset of Figure 2b presents a sensitivity analysis, showing how *R*^2^ varies with parameters *A* and *B*, with the optimal values found to be *A* = 1.18 and *B* = 0.51. Note that the best-fit values of *A* and *B* differ from a Newtonian fluid, *A* = 7.3 and *B* = 0.703. The lower value of *B* for non-Newtonian fluids reflects a weaker dependence of θ_D_ on Ca. One possible explanation for the difference in parameters is the reduced viscous bending of the interface near the contact line due to the shear-thinning viscosity of the fluids, as shown by Seevaratnam et al.^3^

The general scaling result shown in Figure 2b was previously alluded to by Charitatos et al. for spreading in a curtain-coating process.^35^ Although their geometries are different, the authors found through numerical simulations that an effective viscosity could generalize the dynamic contact angle for polymer solutions. However, the effective viscosity proposed is not easily determined and requires a characteristic length scale that is dependent on the shape of the interface. In our work, the effective viscosity is readily captured by an average viscosity calculated by using fluid properties and rheology. This value can be calculated a priori and thus used to make predictions of fluid spreading. Note that we also tested other approaches to determining an effective viscosity such as using the zero-shear rate viscosity, μ_0_, the high shear rate viscosity, μ_∞_, and the solvent viscosity, μ_solvent_. However, they did not result in a generalized collapse of the dynamic contact angle with Ca. More details are presented in the respective section in Supporting Information S3 (Figure S5).

It is reasonable to conclude that eq 1 with modified Ca and μ_avg_ serves as a generalized dynamic contact angle model, as it successfully correlates a wide range of non-Newtonian shear-thinning fluids with varying rheological properties and wetting conditions. Figure 2b shows that the experimental data align well across the range of Ca numbers from 0.001 to 0.1. However, there is slightly more variability between experimental points at Ca > 0.01, which correspond to the early stages of droplet spreading. Therefore, it is possible that this dynamic contact angle model (eq 1) is applicable only to low Ca. However, this should not affect the model’s ability to predict the droplet spreading process, as the early stage is primarily driven by gravitational forces on the fluid bulk rather than edge surface tension forces. The observed master curve is a very surprising result given that all of the fluids have very different shear thinning behavior and are represented by different constitutive models. The theoretical reason why all of the curves collapse when considering a single average viscosity for each fluid is not presently clear to the authors. However, the following explanations may qualitatively describe this process. In the early stage of spreading, the droplet experiences inertia-driven spreading dictated by its bulk properties.^9^ During the later stage, energy dissipation near the contact line and wedge flow primarily controls the wetting dynamics.^52,53^ Consequently, the average shear rate in the bulk governs the early stage, while the average shear rate at the droplet’s edge dominates the later stage. Throughout spreading, shear rates in both the bulk and edge decrease, but the edge region maintains higher shear rates than that of the bulk. This indicates that the governing shear rate remains relatively stable as the dominant influence shifts from the bulk to the edge. This stability may explain why an average viscosity effectively describes the spreading behavior.

**Figure 3:**
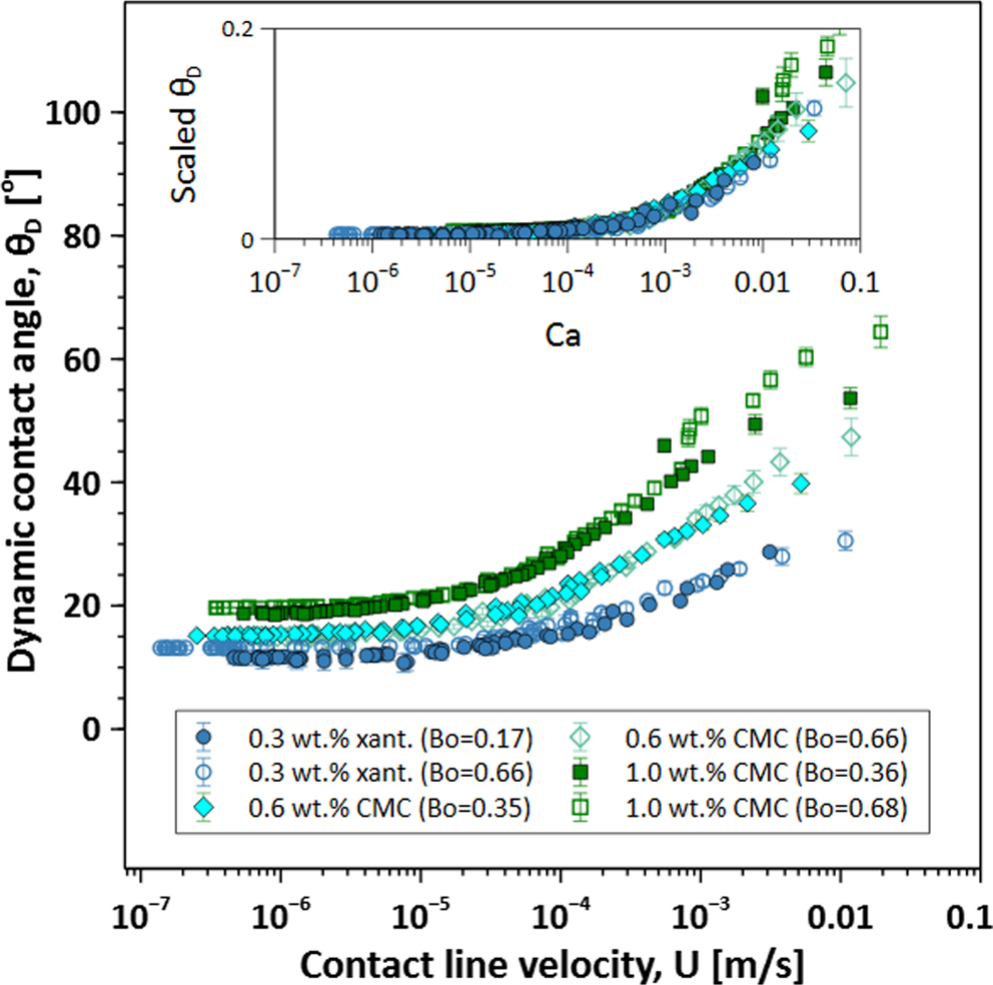
Effect of the droplet size (Bo) on the dynamic contact angle for xanthan and CMC solutions. Filled and hollow points are small and large Bo numbers, respectively. The inset shows the scaled data using the corresponding μ_avg_ for each fluid.

Figure 3 shows θ_D_ as a function of *U* for different-sized droplets of the same fluids. The size of the droplet is an important parameter as it determines the effect of gravity on both the shape and the rate of spreading. The relative magnitude of gravity to interfacial tension is represented by the Bond number, Bo = Δρ*gR*_0_^2^/σ, where Δρ is the difference in density between the fluid and air, *g* is the gravitational constant, and *R*_0_ is the initial radius of the drop before spreading. Previous studies, including dynamic contact angle models based on hydrodynamic theory for power-law shear-thinning fluids, incorporate both the fluid’s rheological properties and the initial droplet size into their formulations.^17,20,53^ Liang et al.^18^ also introduced a modified Ca number accounting for the volume effects for shear-thinning fluids. It has been experimentally shown that for non-Newtonian fluids, the curvature of the moving contact line is directly related to the macroscopic flow geometry, such as *R*_0_, and impacts the dynamic contact angle relation.^54,55^ However, it has been emphasized that for low-concentration polymer solutions, the impact of flow geometry is minimal. Additionally, Min et al.^54^ indicate that the dynamic contact angle remains unaffected by flow geometry at low contact line velocities, i.e., low Ca numbers.^54^ Ngan and Dussan^56^ identified a critical Ca number of 0.005, below which the impact of flow geometry on the observed contact angle is negligible, while Zheleznyi^57^ reported a critical value of 0.05 for Newtonian fluids. Thus, the dynamic contact angle of droplets at low Bo and low Ca numbers can be assumed to be unaffected by droplet geometry.

In this work, we considered only sufficiently small droplets such that the effect of gravity is almost negligible. It is evident in Figure 3 that the dynamic contact angle is not strongly affected by the range of Bo numbers studied in this work, i.e., Bo ≤ 0.7. Therefore, the small Bond number approximation is valid and supports the assumptions in the spreading model described above. The model’s applicability to higher Bond numbers still needs to be tested. The inset of Figure 3 shows the collapse of scaled data using the μ_avg_ and the corresponding Ca number for each fluid and two different droplet radii. It should be noted that our proposed dynamic contact angle model implicitly accounts for the effect of droplet size through the upper limit used to calculate the average viscosity. As mentioned above, γ̇_max_ in eq 3 is inversely proportional to *R*_0_. Increasing the droplet size decreases γ̇_max_, which in turn increases μ_ave_ and Ca. Therefore, the model predicts an increase in the dynamic contact angle with increasing droplet size, which aligns with experimental observations and reports in the literature.^18^

The variability of experimental data at high Ca raises the question regarding the possible effect of geometry on the dynamic contact angle. While most of the Ca measured falls within the small Ca limit for Newtonian fluids, there are some values of Ca that fall above. Incidentally, the largest discrepancy between experimental measurements is in this range for both the inset of Figures 3 and 2b. It is not clear whether the discrepancy is due to an error in evaluating the contact angle, which is largest for θ > 90°, i.e., large Ca, or possibly the effect of geometry on the contact angle, as discussed above. Thus, it is possible that the same limits on Ca for Newtonian fluids hold for non-Newtonian fluids for the effect of geometry on the dynamic contact angle.^54,55^ In any case, these values of Ca represent very early times and are not expected to influence the overall spreading dynamics.

### Spreading Model

**Figure 4:**
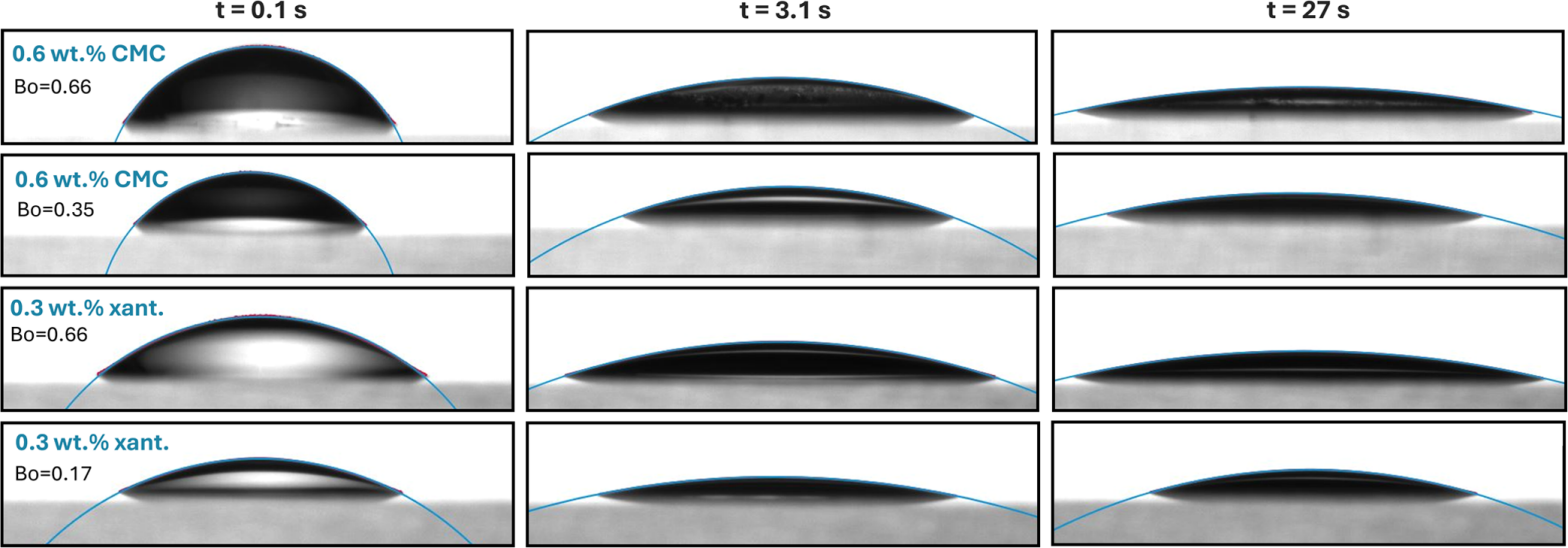
Images of 0.6 wt % CMC and 0.3 wt % xanthan solutions at two different sizes and three distinct times, representing the early, middle, and late stages of spreading. Blue contours show the fitted spheres.

As explained earlier, to predict the spreading radius of shear-thinning fluids, we used the developed dynamic contact angle model (eq 1) along with a spherical cap geometrical argument to correlate θ_D_ to *R*(*t*), i.e., eq 5. To test the spherical gap assumption, images of 0.6 wt % CMC and 0.3 wt % xanthan solutions at three distinct times, representing the early, middle, and late stages of spreading along with the best fit circle to the droplet shapes, are shown in Figure 4. In all cases, the circle fits the interface shape quite well, demonstrating that droplets with Bo ≤ 0.7 are represented by a spherical cap. Note that at very early times, there is a minor inflection point of the contact line, which shows a slight deviation from a circle. However, this inflection is not present in all fluids and is only present at very short times. The inflection is most obvious for fluids of low viscosity and is only observed in the first few hundred milliseconds of spreading. For highly viscous solvents, the inflection point is barely detectable, and the spherical cap model fits the entire spreading process. Figure 4 validates the assumption of eq 5.

**Figure 5:**
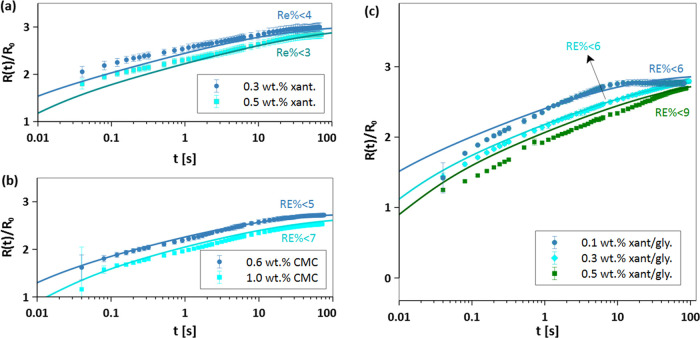
Validation of the developed DAE model: comparison between model and experimental results of (a) Xanthan, (b) CMC, and (c) xanthan/glycerol solutions at different concentrations.

Now that all assumptions of the DAE spreading model are validated; the prediction of the basal radius via eqs 4 and 5 can be directly compared to the experimental data. Figure 5 shows the evolution of the basal radius over time for fluids listed in Table 1, alongside the predictions from the DAE spreading model. The model predicts the evolution of *R*(*t*) very well for xanthan and the CMC in water. The prediction for xanthan in glycerol/water is satisfactory at long times but shows a slight deviation at short times. The model overpredicts the spreading slightly, suggesting that the average viscosity at short times is smaller than the fluid is sampling. We also compared the model predictions to the experimental spreading data reported in the literature to further validate the model. Figure S6 shows excellent agreement between model and experimental data measured by different groups; see Supporting Information S4. These results confirm the validity of our semiempirical DAE spreading model for predicting the basal radius dynamics of non-Newtonian shear-thinning fluids.

**Figure 6:**
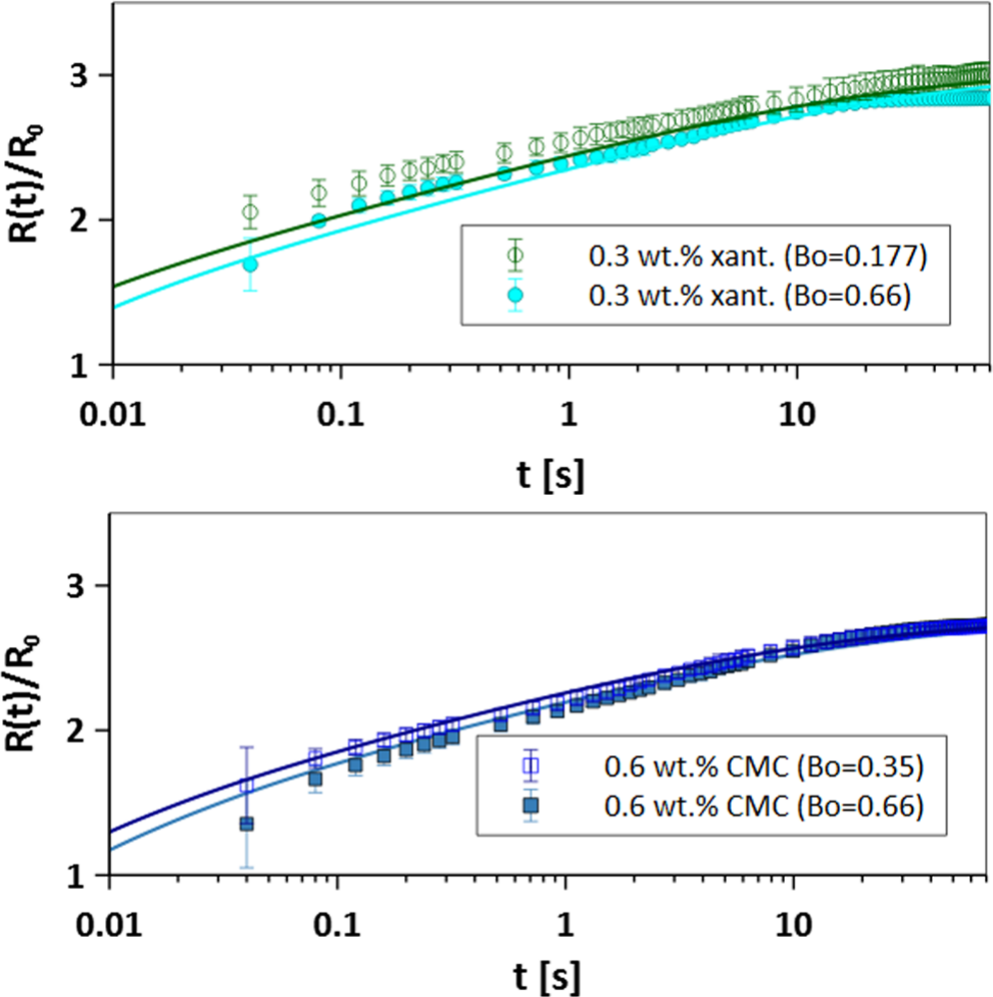
Effect of droplet size, i.e., Bo number of spreading radius: experimental data of xanthan and CMC solutions vs the DAE model prediction.

Although the shape of the droplets can be described by a spherical cap and does not depend on the Bo number, there does exist a dependence of spreading on the size of the droplet. As mentioned above, this effect is due to the larger volume and the dependence of maximum shear rate on radius sampled by the droplet due to the effect of gravity. The DAE model takes into account the initial radius through the droplet volume. Furthermore, the upper limit of integration for the average viscosity (eq 3) depends on the initial size of the drop. Note that as the droplet becomes larger, the maximum shear rate experienced by the droplet decreases, which again comes from an energy balance argument. This is an important effect as it means that the average viscosity is larger for larger droplets, and thus, they should exhibit slower spreading. Figure 6 shows the scaled spreading basal radius for 0.3 wt % xanthan and 0.6 wt % CMC solutions for two different radii. As expected, the larger droplet spreads slower than the smaller droplets. This effect is captured in the definition of the average viscosity, which gives a larger viscosity for a larger starting droplet size due to the difference in the initial shear rate (γ̇_max_).

**Figure 7:**
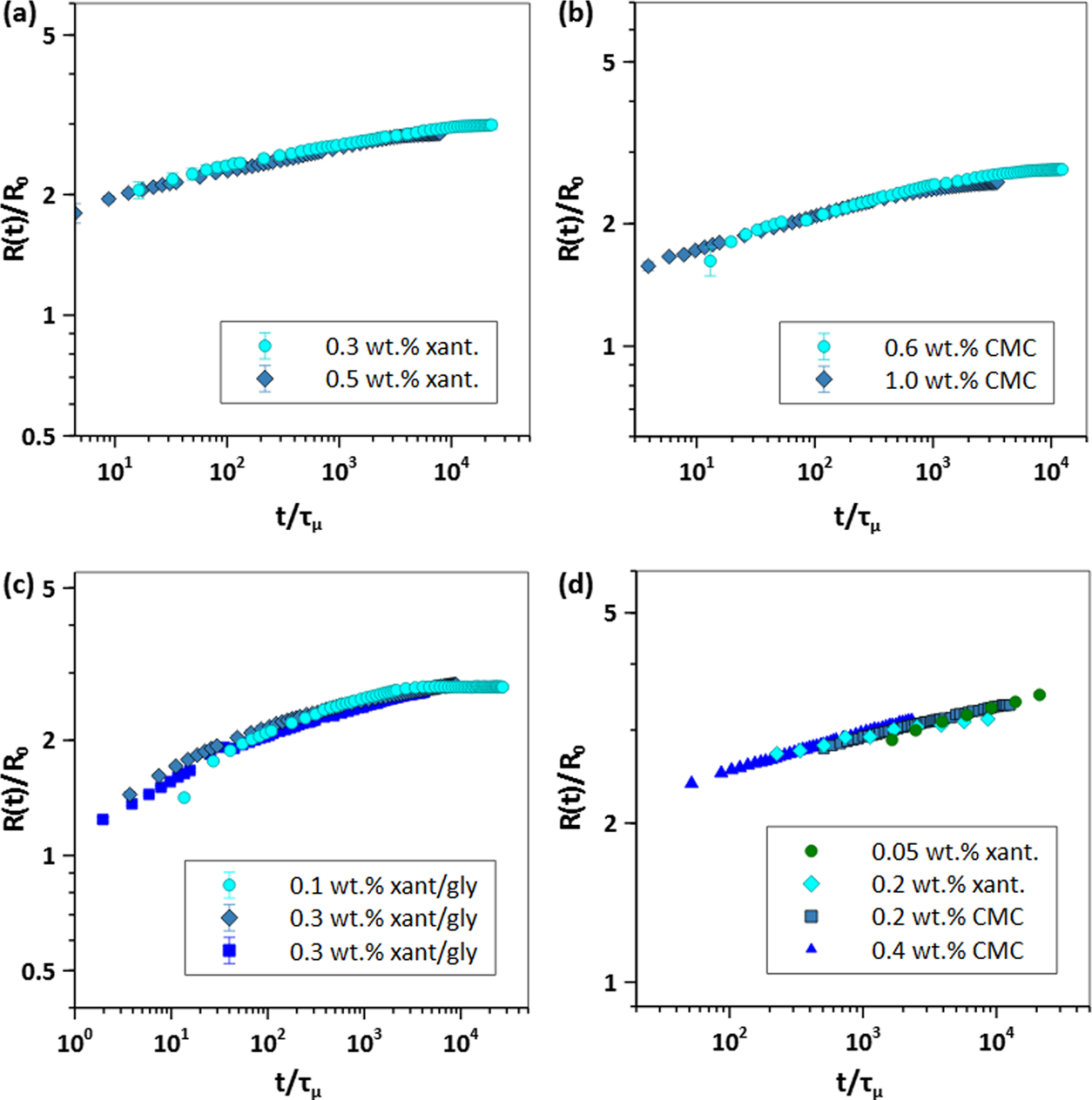
Spreading master curve for non-Newtonian fluids using the viscous time scale defined as τ_μ_ = μ_avg_*R*_0_/σ, for (a) θ_s_ = 13°, (b) θ_s_ = 10°, (c) θ_s_ = 15°, and (d) θ_s_ = 0°.

Previous research has demonstrated that for Newtonian droplets, scaling the spreading time, *t*, with the viscous time scale, τ_μ_ = μ*R*_0_/σ, and scaling the basal radius, *R*(*t*), with *R*_0_, generate a master spreading curve at a given Bo and θ_s_.^9^ Increasing θ_s_ slows down the spreading, and increasing Bo enhances the spreading. In this study, we examine the concept of the spreading master curve for shear-thinning fluids using the proposed μ_avg_ for each fluid. We define the viscous time scale as τ_μ_ = μ_avg_*R*_0_/σ and use it to scale the spreading time. Note that having similar θ_s_ and Bo values is important to construct the master curves. All of the fluids reported in Table 1 have similar Bo values ranging from 0.15 to 0.35. We already showed that the effect of Bo ≤ 0.7 on the spreading of non-Newtonian fluids is minimal. Figure 7a–c shows the scaled spreading curves for each type of fluid shown in Figure 5 using the μ_avg_ reported in Tables 2 and 3. Figure 7d shows the master curves generated for experimental data extracted from the literature of 0.05–0.2 wt % xanthan solutions and 0.1–0.4 wt % CMC solutions on mica with θ_s_ ≃ 0.^21,45^ As shown, similar to Newtonian fluids, for each set of Bo and θ_*s*_, a master spreading curve exists for non-Newtonian shear-thinning fluids. Note that our developed DAE spreading model also shows a master spreading curve using the τ_μ_; see Supporting Information S5 for more details. To verify the significance of the data collapse shown in Figure 7 with respect to the choice of μ_avg_, we also tested different effective viscosities, including (i) zero-shear viscosity, (ii) infinite-shear viscosity, and (iii) solvent viscosity, for each test fluid to construct the spreading master curves. However, they failed to fully represent the spreading process and scale the data across all test fluids; see Figures S6–S8.

## Conclusions

The theoretical and experimental understanding of dynamic wetting has predominantly been restricted to the study of Newtonian fluids. However, many real-world fluids are non-Newtonian, exhibiting complex rheological behaviors, which significantly influence their wetting dynamics. This study introduced a simple spreading model for the dynamic wetting of non-Newtonian shear-thinning fluids. The model is based on the assumption of a low Bond number and a generalized behavior of the dynamic contact angle on the capillary number. The model assumptions were tested against a large dataset of non-Newtonian fluids with different wetting conditions and drop sizes. Surprisingly, the experimental data clearly showed that there exists a general relationship between the dynamic contact angle when the capillary number was defined by an average viscosity. The dependence of the dynamic contact angle on the capillary number was captured by the same model previously used for Newtonian fluids but with different fitting parameters. Undoubtedly, the dynamic contact angle results and scaling arguments outlined herein will be useful in the formulation and testing of inner-scale mechanisms for HD models. The generalized dynamic contact angle dependence was coupled with the equation for the volume of a spherical cap to solve for the spreading radius as a function of time. The model predictions were compared to the experimental spreading data of three fluids with different concentrations, as well as spreading data extracted from the literature, and shown to be in very good agreement. Similar to Newtonian fluids, when experimental time is scaled with a viscous time scale based on the average viscosity, the spreading curves result in a master spreading curve that depends only on the steady-state advancing contact angle. This work provides insights for improving the control and predictability of processes involving the dynamic wetting of dilute polymer solutions such as in additive manufacturing. More work is needed to determine whether this simple model can be applied to higher viscosity samples as well as more complex fluids.

## Supporting Information Available

Dynamic contact angle measurement, average viscosity of shear-thinning fluids, scaling dynamic contact angles with effective viscosity, validation of the semitheoretical DAE spreading model, and master spreading curve for non-Newtonian fluids (PDF)
