# Supplementary material for: Dilute Polymer Droplets Show Generalized Wetting Dynamics via an Average Viscosity
Source: ACS Appl Polym Mater. 2024 Sep 24;6(19):11997–2006. doi: 10.1021/acsapm.4c02170 (PMC11474820; doi:10.1021/acsapm.4c02170)
Supplement: Supplementary file 1 — ap4c02170_si_001.pdf [file ap4c02170_si_001.pdf]

Supporting Information
Dilute Polymer Droplets show Generalized Wetting Dynamics via an Average Viscosity

Amir Azimi Yancheshme, Heedong Yoon, Giuseppe R. Palmese, and Nicolas J. Alvarez*

Chemical and Biological Engineering, Drexel University, Philadelphia, PA 19104, USA

S1 Dynamic contact angle measurement

The dynamic shapes of droplet interfaces were analyzed using Ossila Contact Angle v4.1.4 software by fitting a polynomial curve to the droplet interface locally near the contact line and extending it to the substrate to measure contact angles. The accuracy of the polynomial fits was confirmed by Root Mean Square Error (RMSE) values using Eq. SS1, all below 0.6, indicating excellent fits. For example, Fig. S1 illustrates the polynomial fit on a 0.3 wt.% Xanthan solution in a 60:40 DI-water mixture, along with the RMSE for the left and right contact angles during spreading.

$$RMSE = \sqrt{\frac{1}{n}\sum(y_i - \hat{y}_i)^2} \tag{S1}$$

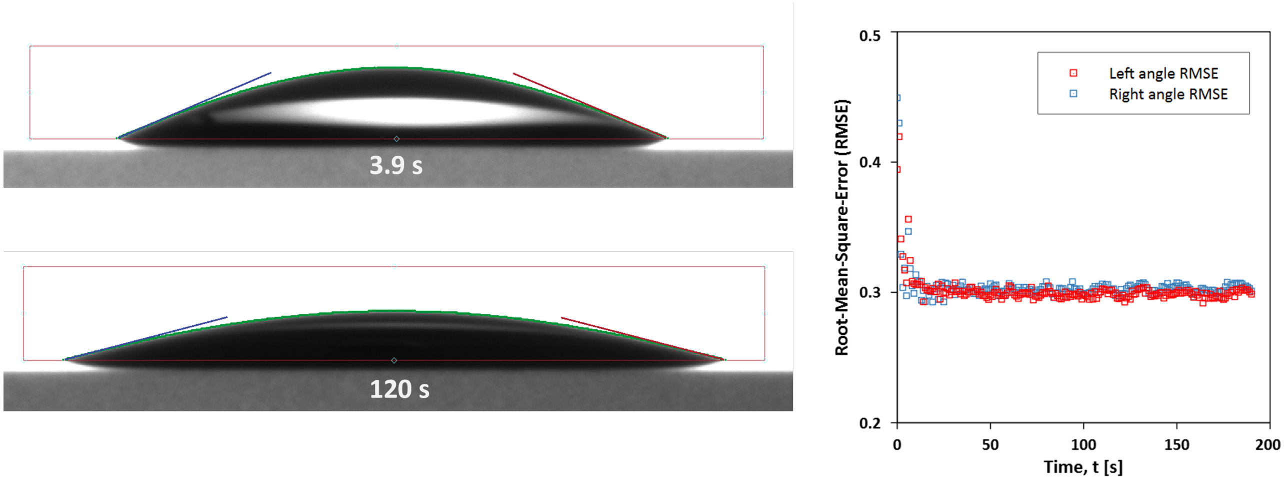

Figure S1: 0.3 wt.% Xanthan in 60:40 DI-water:glycerol solution: tangent lines at the triple points and dynamic RMSE of both left and right angle during the spreading.

The polynomial fit does not account for the effects of gravity on the entire droplet shape, unlike the Young-Laplace fit, meaning that it is not geometry-independent measurement method. In Figure S2, for a 0.3 wt.% xanthan solution, we measured the dynamic contact angle using both the polynomial and Young-Laplace fits. The results are very similar, indicating that the effect of gravity on the dynamic contact angle is negligible, supporting our findings in Figure 3, where we concluded that gravity's influence on the dynamic contact angle in our system can be disregarded.

*Corresponding author: nja49@drexel.edu

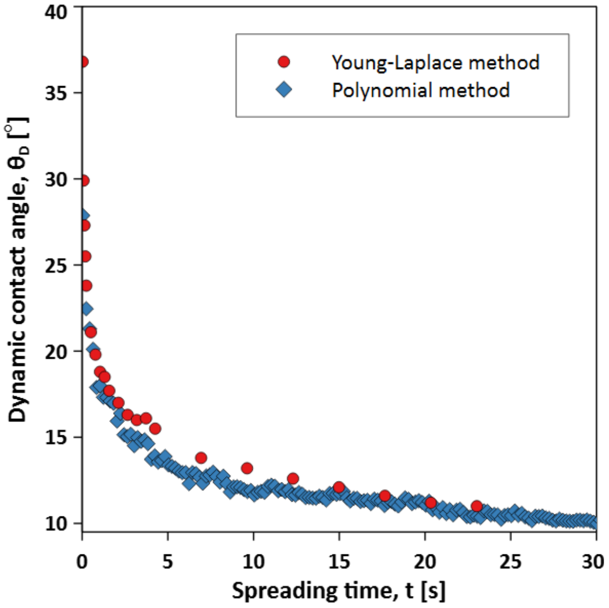

Figure S2: Comparison of dynamic contact angle measurement methods for 0.3 wt.% Xanthan solution: Polynomial vs. Young-Laplace.

S2 Average viscosity of shear thinning fluids

Based on the dynamic contact angle data (θ_D vs U, Fig. 2a), we introduced an average viscosity derived from the fluid's rheology (Eq. 3) over the range $\dot{\gamma}_{min}$=0.01 to $\dot{\gamma}_{max} = (g/2R_0)^{0.5}$ to generalize the dynamic contact angle data. Regarding the lower limit of integral, a sensitivity analysis, shown in Fig. S3, was conducted to assess the impact of varying $\dot{\gamma}_{min}$ from 0.0001 to 10, while keeping $\dot{\gamma}_{max}$ constant. The results indicate that the average viscosity is only weakly dependent on the lower shear-rate cutoff up to around 0.1 s^{-1}, confirming that our chosen lower limit is appropriate.

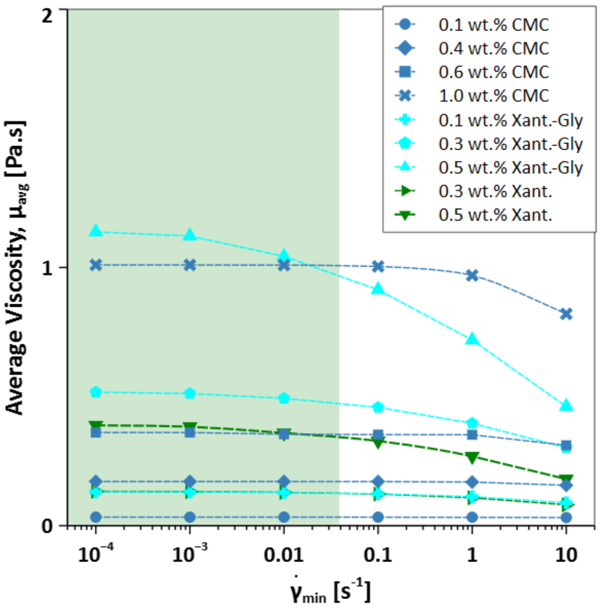

Figure S3: Average viscosity of test fluids using Eq. 3 at different values of lower limit of integral, i.e. $\dot{\gamma}_{min}$.

For the upper limit, the energy balance indicates it is given by $\dot{\gamma}_{max} = (g/2R_0)^{0.5}$. Figure S4 presents the energy balance analysis and the derived shear rate expression at the onset of spreading. Note that the approximation $V_0 = \sqrt{gr_0/2}$ represents the impact velocity experienced by the fluid molecules. To ensure that inertia is negligible in these experiments, we calculated the Weber

number (We $= \rho V_0^2 R_0/\sigma$) for the fluids listed in Table 1. The calculated Weber numbers, ranging from 0.07 to 0.14, indicate that surface tension forces dominate over inertia.

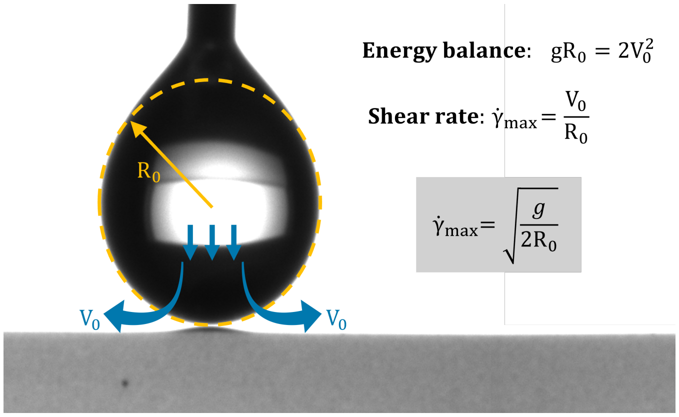

Figure S4: Energy balance analysis to calculate the maximum shear rate at the onset of droplet spreading.

S3 Scaling dynamic contact angles with effective viscosity

We demonstrated that the proposed μ_{avg} effectively collapses the dynamic contact angle data onto a single master curve (Fig. 2b). To evaluate other possible effective viscosities for scaling, we tested various options including zero-shear, infinite-shear, and solvent viscosities for each fluid. These constant viscosities, listed in Table S1, were used to scale the dynamic contact angle data, as shown in Fig. S5. However, none of these alternatives provided a better data collapse than the average viscosity proposed in this study (Eq. 3).

**Fluid**	μ_0 **[Pa.s]**	μ_∞ **[Pa.s]**	$\mu_{solvent}$ **[Pa.s]**
0.05 wt.% xant. [2]	0.0653	0.0011	0.001
0.2 wt.% xant. [2]	1.5488	0.0012	0.001
0.3 wt.% xant.	91.8073	0.0014	0.001
0.5 wt.% xant.	396.3406	0.002	0.001
water/gly+0.1 wt.% xant.	50.9339	0.01	0.018
water/gly+0.3 wt.% xant.	362.8297	0.02275	0.018
water/gly+0.5 wt.% xant.	989.359	0.0285	0.018
0.1 wt.% CMC [1]	0.049	0.001	0.001
0.4 wt.% CMC [1]	0.3382	0.001	0.001
0.6 wt.% CMC	1.11	0.001	0.001
1.0 wt.% CMC	6.515	0.001	0.001

Table S1: zero-shear viscosity, infinite-shear viscosity, and solvent viscosity of test fluids.

S4 Validation of semi-theoretical DAE spreading model

To further validate our developed DAE spreading model, represented by Eqs 4-5, we compared the model's predictions with experimental data from 0.05-0.2 Xanthan solutions [2] and 0.1-0.4 CMC solutions [1], as shown in Fig. S6. The detailed properties of the fluids and the wetting conditions are reported in Table 1 of the main manuscript.

S5 Master spreading curve for non-Newtonian fluids

We showed that non-Newtonian fluid spreading data forms a master curve when the spreading time is scaled using the viscous timescale, defined by the average viscosity, $\tau_\mu = \mu_{avg} R_0/\sigma$, for a given Bo and θ_s. To confirm the robustness of this data

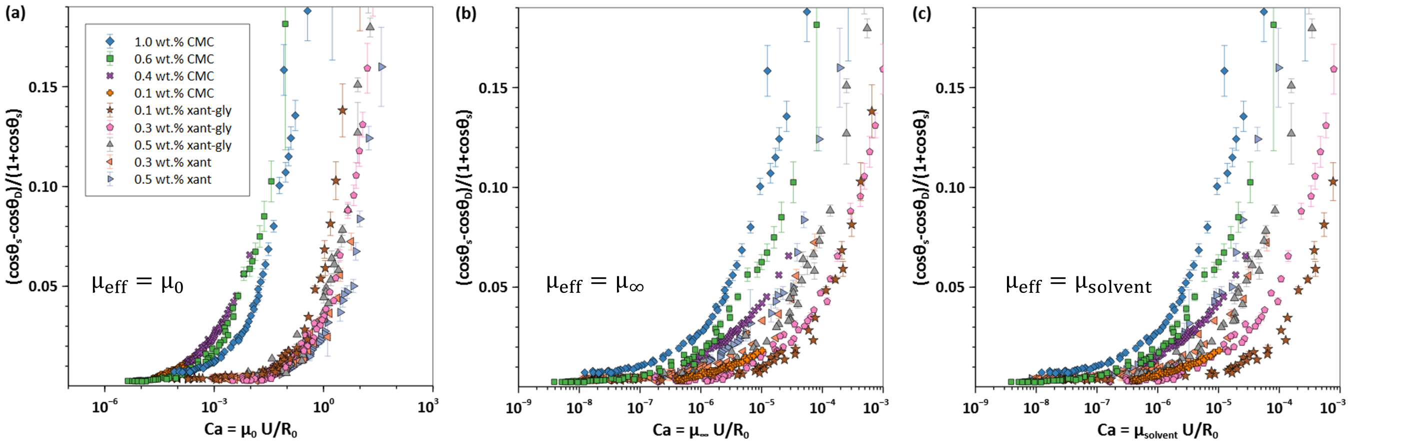

Figure S5: Scaled dynamic contact angle vs capillary number defined using a) zero-shear μ, infinite-shear μ, and solvent μ.

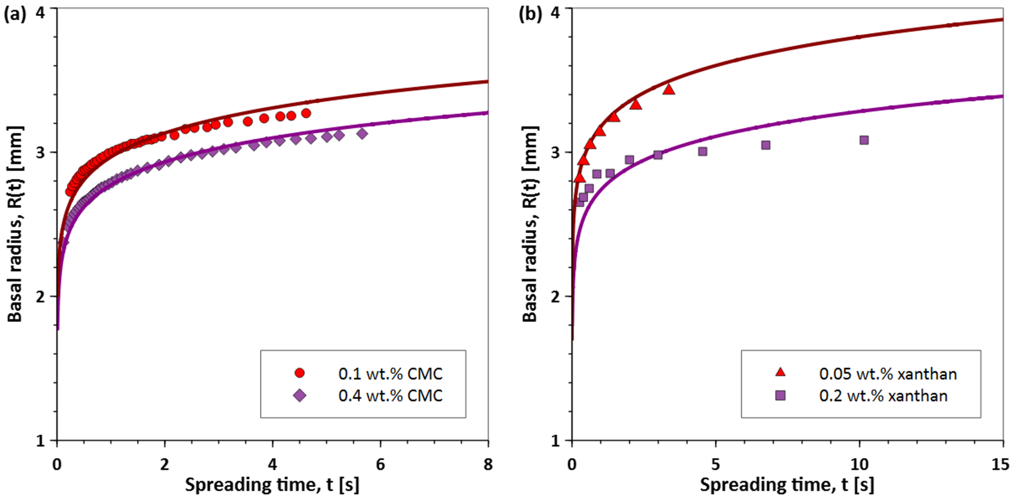

Figure S6: DAE spreading model (lines) versus experimental data (dots) for **a)** CMC solutions [1], **b)** Xanthan solutions [2].

collapse, we tested different characteristic viscosities, including zero-shear, infinite-shear, and solvent viscosities, and used them to define the viscous timescale for scaling the spreading data. As demonstrated in Figs. S7-S9, these constant viscosities fail to fully represent the spreading process and scale the data across all test fluids, in contrast to our proposed μ_{avg}, which successfully collapses all the sampled data.

we also examine the concept of a spreading master curve using our developed DAE model with the proposed average viscosity, μ_{avg}. As shown in Fig. S10, the model predicts the spreading behavior of various shear-thinning fluids with different average viscosities, all sharing the same $\mathrm{Bo} = 0.3$ and $\theta_s = 0$. The inset illustrates that scaling the spreading time by τ_μ, defined with μ_{avg}, produces a master curve. Converting DAE spreading model (Eqs. 5-6) to a non-Dimensional form using $\bar{R} = R/R_0$ and $\bar{t} = t/\tau_\mu$, where $\tau_\mu = \mu_{avg} R_0/\sigma$, would results in:

$$\frac{d\bar{R}}{d\bar{t}} = \left(\frac{1}{A} tanh^{-1}\left(\frac{cos\theta_s - cos\theta_D}{1 + cos\theta_s}\right)\right)^{\frac{1}{B}} \tag{S2}$$

$$4 = \bar{R}^3 \left(\frac{2 - 3\cos(\theta_D) + \cos^3(\theta_D)}{\sin^3(\theta_D)}\right), \tag{S3}$$

Which are only depend on the θ_s and confirms that the model can generate master spreading curves using the viscous timescale.

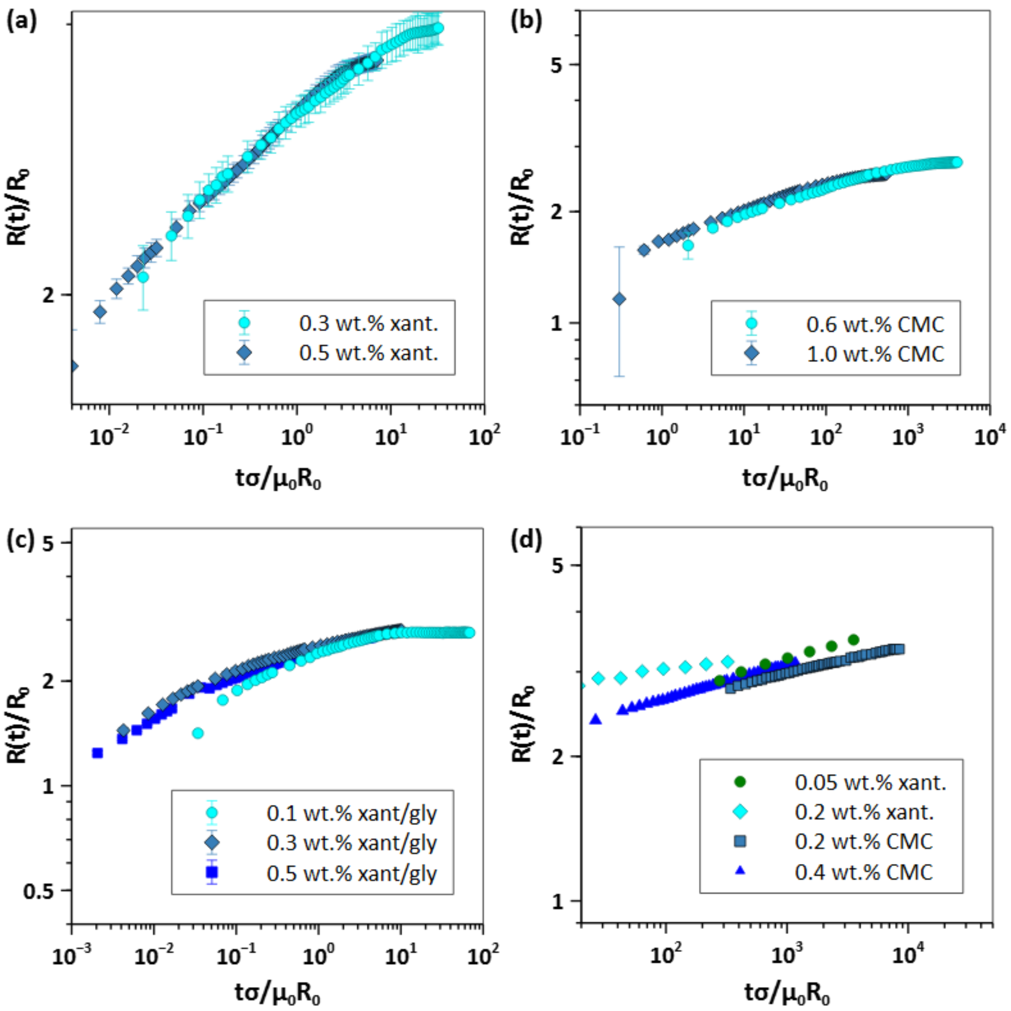

Figure S7: Scaled spreading curves for non-Newtonian fluids using viscous timescale defined as $\tau_\mu = \mu_0 R_0/\sigma$, for **a)** $\theta_s = 13°$, **b)** $\theta_s = 10°$, **c)** $\theta_s = 15°$, and **d)** $\theta_s = 0°$.

References

[1] Qi Min et al. "Spreading of completely wetting, non-Newtonian fluids with non-power-law rheology". In: *J. Colloid Interface Sci.* 348.1 (2010), pp. 250–254.

[2] XD Wang et al. "Spreading of completely wetting or partially wetting power-law fluid on solid surface". In: *Langmuir* 23.18 (2007), pp. 9258–9262.

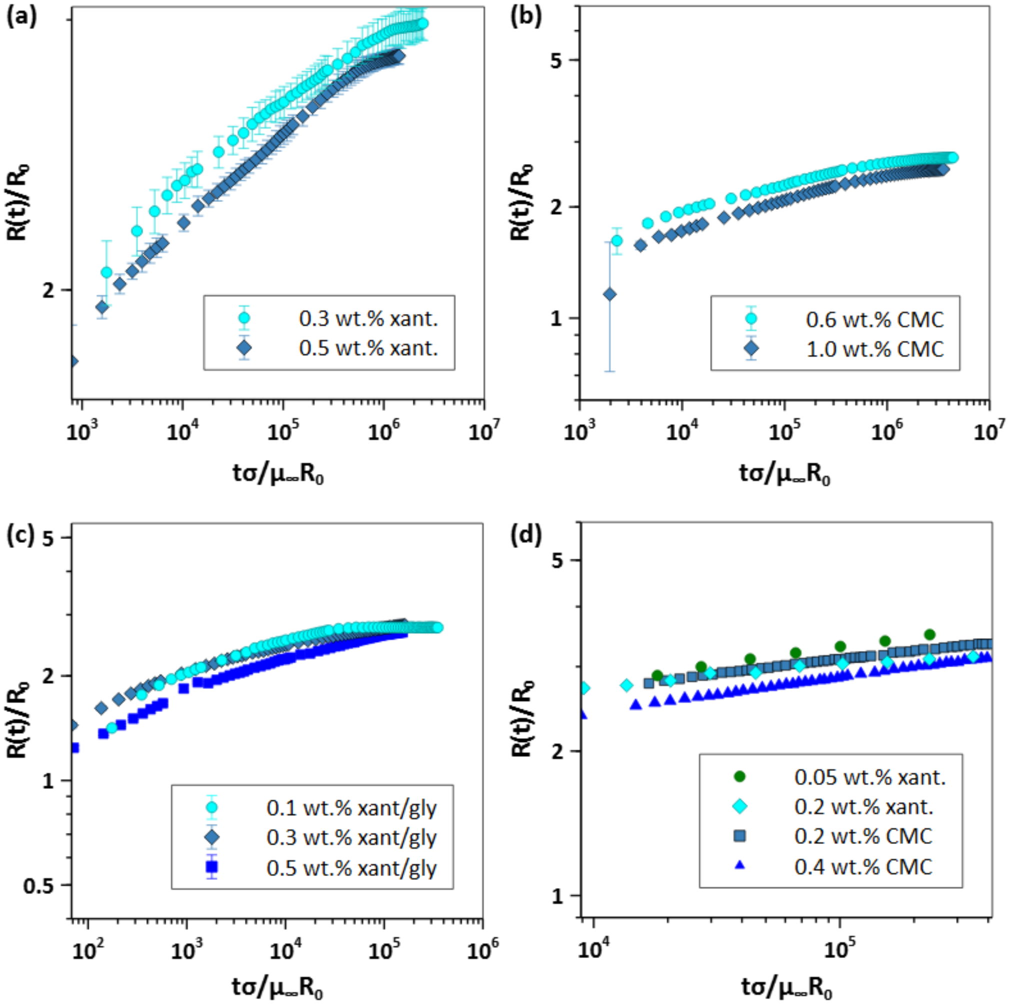

Figure S8: Scaled spreading curves for non-Newtonian fluids using viscous timescale defined as $\tau_\mu = \mu_\infty R_0/\sigma$, for **a)** $\theta_s = 13°$, **b)** $\theta_s = 10°$, **c)** $\theta_s = 15°$, and **d)** $\theta_s = 0°$.

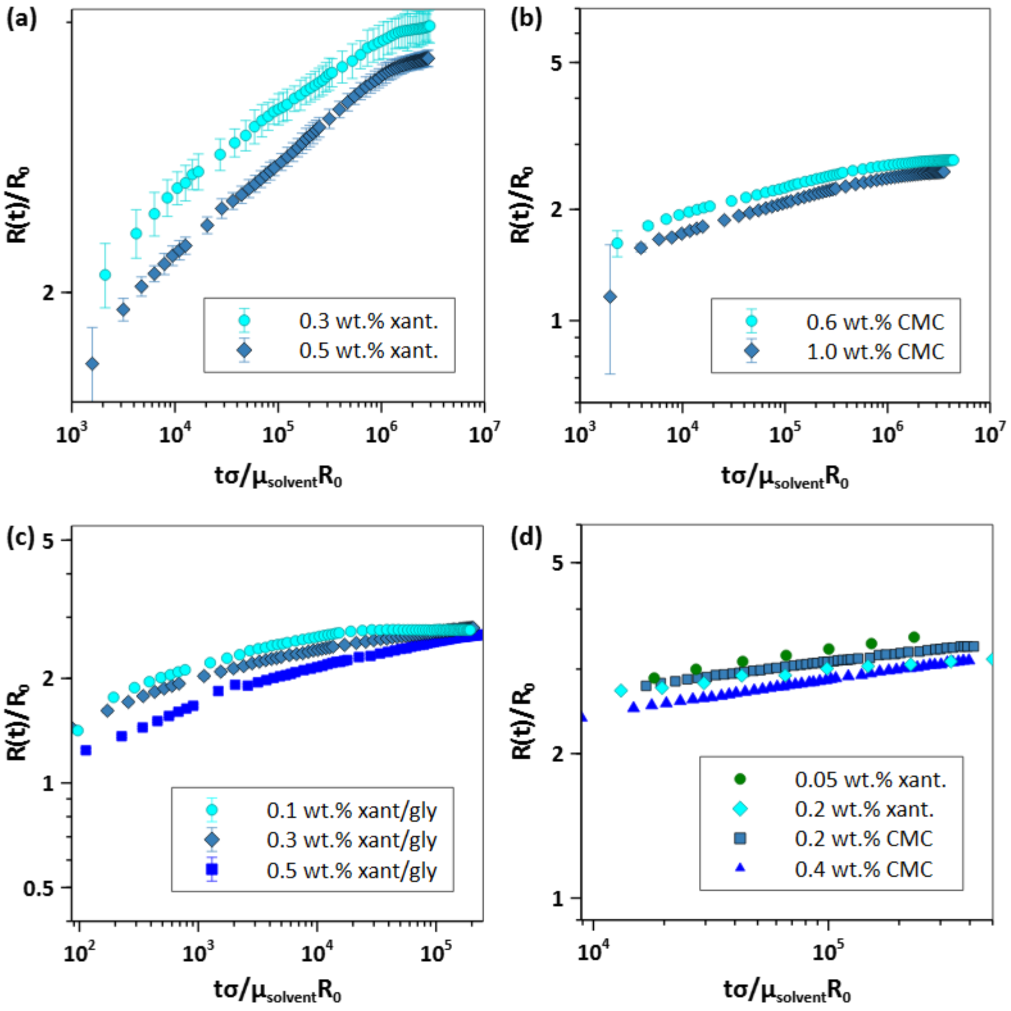

Figure S9: Scaled spreading curves for non-Newtonian fluids using viscous timescale defined as $\tau_\mu = \mu_{solvent} R_0/\sigma$, for **a)** $\theta_s = 13°$, **b)** $\theta_s = 10°$, **c)** $\theta_s = 15°$, and **d)** $\theta_s = 0°$.

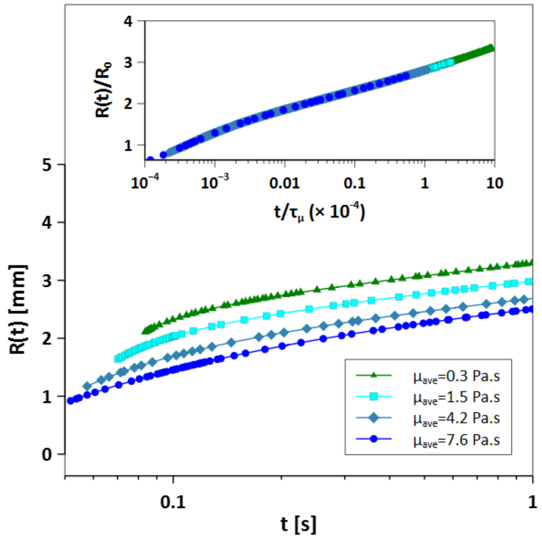

Figure S10: Spreading mastercurve for non-Newtonian fluids using viscous timescale defined as $\tau_\mu = \mu_{avg} R_0/\sigma$ at a fixed Bo $= 0.3$ and $\theta_s = 0$
